# The *IL-1B* Gene Polymorphisms rs16944 and rs1143627 Contribute to an Increased Risk of Coronary Artery Lesions in Southern Chinese Children with Kawasaki Disease

**DOI:** 10.1155/2019/4730507

**Published:** 2019-04-09

**Authors:** Lan Yan Fu, Xiantao Qiu, Qiu Lian Deng, Ping Huang, Lei Pi, Yufen Xu, Di Che, Huazhong Zhou, Zhaoliang Lu, Yaqian Tan, Zhiyong Jiang, Li Zhang, Techang Liu, Xiaoqiong Gu

**Affiliations:** ^1^Department of Clinical Biological Resource Bank, Guangzhou Institute of Pediatrics, Guangzhou Women and Children's Medical Center, Guangzhou Medical University, Guangzhou, 510623 Guangdong, China; ^2^Department of Clinical Lab, Guangzhou Institute of Pediatrics, Guangzhou Women and Children's Medical Center, Guangzhou Medical University, Guangzhou, 510623 Guangdong, China; ^3^Department of Cardiology, Guangzhou Women and Children's Hospital, Guangzhou Medical University, Guangzhou, 510623 Guangdong, China; ^4^Department of Blood Transfusion, Guangzhou Women and Children's Medical Center, Guangzhou Medical University, Guangzhou, China

## Abstract

**Background:**

Kawasaki disease (KD) is a systemic form of self-limited vasculitis in children less than five years old, and the main complication is coronary artery injury. However, the etiology of KD remains unclear. The *IL-1B* polymorphisms rs16944 GG and rs1143627 AA and their diplotype GA/GA have been associated with significantly increased risk of intravenous immunoglobulin (IVIG) resistance in a Taiwanese population, but the relationship between rs16944 A/G and rs1143627 G/A and coronary artery lesions (CALs) in patients with KD has not been investigated. The present study is aimed at investigating whether the rs16944 A/G and rs1143627 G/A polymorphisms in *IL-1B* were associated with KD susceptibility and CALs in a southern Chinese population.

**Methods and Results:**

We recruited 719 patients with KD and 1401 healthy children. Multiplex PCR was used to assess the genotypes of single nucleotide polymorphisms (SNPs), including two SNPs of *IL-1B*, rs16944 A/G and rs1143627 G/A. According to the results, no significant association was observed between the *IL-1B* (rs16944 and rs1143627) polymorphisms and KD risk in the patients compared with the healthy controls in our southern Chinese population. However, in further stratified analysis, we found that children younger than 12 months with the rs16944 GG and rs1143627 AA genotypes of *IL-1B* had a higher risk of CALs than those with the AA/AG genotypes of rs16944 and GG/AG genotypes of rs1143627 (OR = 2.28, 95% CI = 1.32-3.95, *P* = 0.0032, adjusted OR = 2.33, 95% CI = 1.34-4.04, *P* = 0.0027).

**Conclusions:**

Our results indicated that there was no association between the rs16944 A/G and rs1143627 G/A gene polymorphisms and KD susceptibility. However, the rs16944 GG and rs1143627 AA genotypes of *IL-1B* may significantly impact the risk of CAL formation in children younger than 12 months, which may contribute to the pathogenesis of KD. These findings need further validation in multicenter studies with larger sample sizes.

## 1. Introduction

Kawasaki disease (KD) is characterized by systemic vasculitis and always occurs in children younger than 5 years. KD is also known as mucocutaneous lymph node syndrome [1]. Coronary artery lesions (CALs) are a major complication. In the acute stage, administration of a single high dose of intravenous immunoglobulin (IVIG) is an effective treatment that reduces the incidence of CALs. However, approximately 3-5% of treated children still develop coronary artery abnormalities and coronary aneurysms (CAAs) [2]. Therefore, KD has become the leading cause of acquired heart disease in children and is also an important cause of coronary artery injury in adults [3–5]. Thus far, over 60 countries throughout the world have reported cases of KD; the number of cases is highest in Japan, where the annual incidence rate of KD is approximately 300/100,000 among children less than 4 years old and 10/1,000 have a history of KD by 10 years of age [6–9]. Taiwan of China has the third highest incidence of KD in the world after Japan and Korea, with an incidence of 82.8/100,000 [10, 11]. The etiology of KD is not yet fully understood and may be related to infection, immune response, and genetic susceptibility.

Many studies have shown that immune activation and secretion of various cytokines play a key role in the pathogenesis of KD by mediating the imbalance of proinflammatory and anti-inflammatory responses. A variety of proinflammatory and anti-inflammatory cytokines have been reported to increase significantly during acute KD, such as *IL-1*, *TNF-α*, *IL-6*, *IL-8*, and *IL-10* [12–14]. These proinflammatory cytokines induce endothelial cell apoptosis, which is the cause of vascular endothelial injury in KD and has been implicated in the development of the disease [15–17]. Studies have indicated that genetic abnormalities affect the expression of cytokines, and changes in single nucleotide polymorphisms (SNPs) in genes may influence the function of the corresponding cytokines [18]. The *IL-1* family includes *IL-1α*, *IL-1B*, and *IL-1Ra*, which play fundamental roles in the inflammatory processes of KD. Two SNPs of *IL-1B* with functional implications have been reported, *IL-1B* rs16944 G and *IL-1B* rs1143627 A, and their effects on gene expression have been examined. *IL-1B* rs16944 G has been shown to have a relationship with increased transcriptional activity, rs1143627 A has been found to be related to reduce promoter activity, and the haplotype GA (rs16944 and rs1143627) has been associated with greater transcriptional activity than the other haplotypes. Weng et al. [19] demonstrated that the *IL-1B* rs16944 GG and *IL-1B* rs1143627 AA genotypes or the GA/GA diplotype may be associated with initial IVIG treatment failure in Taiwanese children with KD, but no association with susceptibility to KD was observed. SNPs of *IL-1B* (rs1143634, rs16944, and rs1143627) or *IL-1A*-889 have been reported to have no significant association with KD susceptibility in Korean [20], Iranian [21], or Taiwanese populations [22].

Although polymorphisms in the proinflammatory cytokine *IL-1B* have been investigated in Korean, Iranian, and Taiwanese populations with KD, none has been examined in southern Chinese children with KD. The purpose of this study was to investigate the association of genetic polymorphisms in cytokine *IL-1B* rs16944 A/G and rs1143627 G/A with susceptibility to KD with or without CALs in southern Chinese children.

## 2. Materials and Methods

### 2.1. Study Design

A case control study was conducted on 719 patients with KD at Guangzhou Women and Children Medical Center in China, mainly between February 2013 and November 2017. The diagnosis of KD was based mainly on the Japanese diagnostic criteria [23]. Simultaneously, 1,401 age- and gender-matched subjects without cardiovascular risk factors and fever were selected as a control group. This study was approved by the Guangzhou Women and Children Medical Center Ethics Committee (ethics number: 2014073009) under Trial Registration Number Chi CTR-EOC-1701326. All parents of the patients and control candidates were given detailed information about the study aim and signed informed consent.

### 2.2. DNA Extraction and Genotype

All collected experimental whole blood samples were thawed on ice, and DNA was extracted from 200 *μ*l of whole blood per sample using a Genomic DNA Extraction Kit (Tiangen, Beijing, China) according to the manufacturer's instructions. The concentration and quality of genomic DNA were measured using a nucleic acid quantifier, and the sample was stored at -80°C until later use. We performed multiplex PCR to genotype the SNPs, including rs16944 A/G and rs1143627 G/A. The primer sequences were as follows: rs16944: forward 5′-TAAATGGGTACAATGAAGGGCCA-3′, reverse 5′-CAATTTTCTCCTCAGAGGCTCCT-3′; rs1143627: forward 5′-TCGAAGAGGTTTGGTATCTGCC-3′, reverse 5′-GCTTCCACCAATACTCTTTTCCC-3′. Briefly, high-quality genomic DNA samples were genotyped by PCR using multiple gene-specific primer pairs to enrich the specific SNPs and indexing primers to enable massive parallel sequencing on the Ion Proton System (Life Technologies). For the specific procedures, please refer to our previous article [24]. Moreover, to ensure the accuracy of the genotyping results, we randomly selected approximately 5% of the control and case samples for repeated analysis, and the results were 100% concordant with the initial analysis.

### 2.3. Statistical Analysis

The chi-square test was performed to evaluate the distributions of demographic variables and genotype frequencies in KD patients and controls. Hardy-Weinberg equilibrium (HWE) was calculated for samples by using the chi-squared goodness-of-fit test. The association between the rs16944 A>G and rs1143627 G>A polymorphisms of *IL-1B* and KD susceptibility was evaluated by calculating the odds ratio (OR) and the 95% confidence interval (CI), and an unconditional univariate logistic regression analysis was performed. Adjusted ORs were calculated by multivariate analysis with adjustment for age and gender. All statistical analyses were conducted using SAS software (Version 9.1; SAS Institute, Cary, NC, USA), and *P* < 0.05 indicated statistical significance.

## 3. Results

### 3.1. Clinical Characteristics of Patients with KD

The clinical characteristics are summarized in Table 1. The clinical and demographic variables are from the recruited study population of 719 cases and 1,401 KD-free controls. There were no significant differences between the KD patients and controls in terms of age (*P* = 0.147) and gender (*P* = 0.546). The mean ages were 28.96 ± 25.34 months for patients (range 1-166) and 28.05 ± 28.05 months for controls (range 1-144). Of the KD patients, 32.13% and 67.87% were female and male, respectively, and the controls were 33.55% female and 66.45% male. According to the American diagnostic guidelines, CALs were defined as coronary vessels with an internal diameter ≥ 2.0-3.0 mm in a child younger than 5 years of age or >4.0 mm in those 5 years of age and older [25]. According to the coronary artery condition, the KD patients were divided into those with CALs (43.39%) and without CALs (NCALs) (56.61%).

### 3.2. Associations of IL-1B Gene Polymorphisms with KD Risk and CALs of KD

The genotype distributions of the selected SNPs of *IL-1B*, rs16944 A/G and rs1143627 G/A, and their associations with KD risk are displayed in Table 2. The genotype frequencies of the samples met HWE. Unfortunately, we did not observe any significant associations between the two SNPs and the risk of KD. Using the rs16944 AA genotype as the reference, the AG variant genotype (AG vs. AA) had an adjusted OR of 1.2 (95% CI = 0.95-1.51, *P* = 0.120); the GG genotype (GG vs. AA) had an adjusted OR of 1.17 (95% CI = 0.90-1.51, *P* = 0.233). Using the rs1143627 GG genotype as the reference, the AG variant genotype (AG vs. GG) had an adjusted OR of 1.21 (95% CI = 0.97-1.53, *P* = 0.091), and the AA genotype (AA vs. GG) had an adjusted OR of 1.19 (95% CI = 0.92-1.54, *P* = 0.192). Under the additive, dominant, and recessive models, there were no significant associations between the rs16944 A/G and rs1143627 G/A polymorphisms and KD susceptibility after adjusting for age and gender (rs16944 additive model: adjusted OR = 1.08, *P* = 0.260; dominant model AG+GG vs. AA: adjusted OR = 1.19, *P* = 0.118; recessive model GG vs. AA+AG: adjusted OR = 1.03, *P* = 0.758; and rs1143627 additive model: adjusted OR = 1.09, *P* = 0.210; dominant model AG+AA vs. GG: adjusted OR = 1.21, *P* = 0.087; and recessive model AA vs. GG+AG: adjusted OR = 1.04, *P* = 0.718).

We then assessed whether there were associations between the *IL-1B* gene polymorphisms and susceptibility to CALs in KD. Overall, 312 CALs and 407 NCALs were successfully genotyped, as listed in Table 3. We did not observe any significant associations between the two SNPs (rs16944 A/G, rs1143627 G/A) and the development of KD susceptibility (rs16944 AG vs. AA: adjusted OR = 1.01, *P* = 0.941; GG vs. AA: adjusted OR = 1.27, *P* = 0.277; additive model: adjusted OR = 1.13, *P* = 0.247; rs16944 dominant model AG+GG vs. AA: adjusted OR = 1.10, *P* = 0.616; recessive model GG vs. AA+AG: adjusted OR = 1.25, *P* = 0.179; rs1143627 AG vs. GG: adjusted OR = 1.01, *P* = 0.945; AA vs. GG: adjusted OR = 1.26, *P* = 0.291; additive model: adjusted OR = 1.13, *P* = 0.266; rs1143627 dominant model AG+AA vs. GG: adjusted OR = 1.09, *P* = 0.633; recessive model AA vs. GG+AG: adjusted OR = 1.25, *P* = 0.194) after adjusting for age and gender.

### 3.3. Stratification Analysis of IL-1B Gene Polymorphisms with CAL Susceptibility

We further explored the association between *IL-1B* gene polymorphisms and CALs in children with KD in stratified analyses considering age and gender (Tables 4 and 5). We found that younger children (≤12 months old) with rs16944 GG genotypes and rs1143627 AA genotypes were at significantly higher risk of CALs than those with AA/AG genotypes and GG/AG genotypes (OR = 2.28, 95% CI = 1.32-3.95, *P* = 0.0032, adjusted OR = 2.33, 95% CI = 1.34-4.04, *P* = 0.0027).

## 4. Discussion

In the present study, our results revealed no association between the two selected SNPs in *IL-1B* and KD susceptibility in southern Chinese children, as observed previously in Iranian and Taiwanese populations. We failed to find any significant association between the *IL-1B* (rs16944 and rs1143627) gene polymorphisms and the risk of CALs compared with NCALs in KD. However, in the stratified analysis, if the age of onset was 12 months or younger, we observed that carriers of the *IL-1B* rs16944 GG genotypes and *IL-1B* rs1143627 AA genotypes had a higher risk of CALs in KD than those carrying the *IL-1B* rs16944 AA/AG genotypes and IL-1B rs1143627 GG/AG genotypes.

KD has been extensively studied in terms of etiology, pathogenesis, treatment, prognosis, and intervention factors, but the pathogenesis of KD has not been clearly elaborated [26–28]. Abnormal activation of the immune system is thought to be a central characteristic of KD. Cytokines and inflammatory mediators interact with each other to magnify the immune effect, eventually leading to the persistence of vascular endothelial cell damage and aggravation. Inflammatory cytokines play an important role in KD. Many reports have illustrated that serum levels of cytokines, including interferon-*γ*, tumor necrosis factor *α*, *IL-27*, *IL-10*, *IL-6*, *IL-4*, *IL-2*, and *IL-1B*, are increased significantly in the acute phase of KD [29, 30]. Characterizing serum cytokine profiles may help predict disease prognosis and target treatment strategies in KD patients. Genetic data have revealed the key role of cytokines in the pathogenesis of KD. For example, in a study with 55 cases and 140 controls, Assari et al. [31] found a positive association of the CC genotype of *IL-4* (-590, 33) and a negative association of the CT genotype at -590 with the risk of KD in an Iranian population. Data from studies in the Taiwanese population support the significant associations of the CC genotype and CC/CC diplotype at *IL-10* (-819, -592) with the risk of KD and a relationship of the G allele frequencies of *IL-10* (-1082) gene polymorphisms with CAA development in KD [32, 33]. These cytokine gene polymorphisms have been found to be associated with KD susceptibility, and some SNPs of cytokine genes affect the expression of cytokines in KD. However, some studies of SNPs in genes encoding cytokines, such as *IL-6* (-636 C/G) [34], the *IL-6* promoter at +162 bp, +168 bp, and -594 bp [35], and *IL-4* (-590 C/T, 8375 A/G) [36], have clarified that they have no association with susceptibility to KD.

The *IL-*1 family includes *IL-1α*, *IL-1B*, and *IL-1Ra*, which play fundamental roles in the inflammatory processes of KD. Lee et al. [37] indicated that in a KD mouse model, *IL-1B* regulates the development of CALs and is blocked by an *IL-1* receptor antagonist. Furthermore, *IL-1* levels may be influenced by *IL-1* polymorphisms. Thus, *IL-1A*, *IL-1B*, and *IL1RN* are considered attractive candidate genes for vasculitis. *IL-1B* has been reported to be related to the functionality of SNPs within the gene, and *IL-1B* rs16944 and rs1143627 are essentially in complete linkage disequilibrium [38]. In genetic studies, Weng et al. [19] reported that the haplotypes of *IL-1B* (rs16944 and rs1143627) did not correlate with the risk of KD and IVIG resistance, but the *IL-1B* rs16944 GG and rs1143627 AA genotypes or the GA/GA diplotype significantly increased the risk of IVIG resistance in Taiwanese children with KD. Assari et al. [21] showed no statistically significant association between *IL-1B* (rs16944 and rs1143634) polymorphisms and KD patients in an Iranian population. Furthermore, the results of a study by Kim et al. [20] suggested that *IL-1B* rs1143634 G/A is associated with genetic susceptibility to KD and that there is no significant difference in the frequency of this genotype between KD with CALs (*n* = 32) and KD without CALs (*n* = 77). In the present case control study, we repeated this exploration of *IL-1B* rs16944 and rs1143627 genetic polymorphisms and KD susceptibility, but we also investigated the association of these two SNPs with or without CAL formation in southern Chinese children with KD. We found that two SNPs of the *IL-1B* gene were not associated with KD or the development of CAL susceptibility, but in children less than 12 months of age, compared with carriers of the AA/AG and GG/AG genotypes, carriers of the *IL-1B* rs16944 GG and rs1143627 AA genotypes had a significantly increased risk of development of CALs (*P* = 0.0027), which may be ascribed to the fact that young children may be more genetically susceptible to KD risk. Additionally, the incidence of KD tends to be higher in children younger than 5 years of age. Moreover, according to the data from epidemiological studies, KD is an age- and gender-related disease that generally occurs in children aged <5 years and is more severe in children aged <12 months [39, 40]. However, the factors underlying our results are unclear. There are several limitations that need to be mentioned. First, this was a single-center investigation of southern Chinese children with KD, and thus, the power of the results may be limited. Other centers with larger sample sizes need to be included in replication studies to verify this association. Second, we examined only the *IL-1B* rs16944 A/G and rs1143627 G/A polymorphisms; other potential SNPs of *IL-1B* and potential mechanisms of polymorphisms were not considered and remain to be studied. Third, due to a lack of information on the living environment, exposure factors, and dietary intake, we analyzed only the relationship between *IL-1B* gene polymorphisms and susceptibility to CALs in this study.

In conclusion, although there was no association between *IL-1B* (rs16944 and rs1143627) gene polymorphisms and KD susceptibility or the formation of CALs, these SNPs may contribute greatly to the risk of CALs in southern Chinese children younger than 12 months of age. However, studies investigating the *IL-1B* rs16944 A/G and rs1143627 G/A polymorphisms with multicenter and larger populations are needed to confirm our results.

## Figures and Tables

**Table 1:** Frequency distribution of selected variables for cases and controls.

Variables	Cases (*n* = 719)		Controls (*n* = 1401)		*P ^a^*
	No.	%	No.	%	
Age range, month	1.00-166.0		1.00-144		0.147
Mean ± SD	28.96 ± 25.34		28.05 ± 25.31		
≤12	251	34.91	534	38.12	
12-60	414	57.58	742	53.96	
>60	54	7.51	125	8.92	
Gender					0.546
Female	231	32.13	470	33.55	
Male	488	67.87	931	66.45	
Coronary artery outcomes					
CALs	312	43.39			
NCALs	407	56.61			

CALs: coronary artery lesions; NCALs: no coronary artery lesions. ^a^Two-sided *χ*^2^ test for distributions between cases and controls.

**Table 2:** Genotype distributions of *IL-1B* gene polymorphisms and Kawasaki disease susceptibility.

Genotype	Cases (*N* = 719)	Controls (*N* = 1,401)	*P* ^a^	Crude OR (95% CI)	*P*	Adjusted OR (95% CI)^b^	*P* ^b^
*rs16944* (HWE = 0.34)							
AA	154 (21.42)	342 (24.41)		1.00		1.00	
AG	367 (51.04)	682 (48.68)		1.20 (0.95-1.50)	0.127	1.20 (0.95-1.51)	0.120
GG	198 (27.54)	377 (27.91)		0.24 (0.90-1.51)	0.240	1.17 (0.90-1.51)	0.233
Additive			0.294	1.08 (0.95-1.22)	0.266	1.08 (0.95-1.22)	0.260
Dominant	565 (78.58)	1,059 (75.59)	0.122	1.19 (0.95-1.47)	0.124	1.19 (0.96-1.48)	0.118
Recessive	521 (72.46)	1,024 (73.09)	0.758	1.03 (0.84-1.26)	0.757	1.03 (0.84-1.26)	0.758
*rs1143627* (HWE = 0.47)							
GG	156 (21.70)	350 (24.98)		1.00		1.00	
AG	371 (51.60)	687 (49.04)		1.21 (0.97-1.52)	0.098	1.21 (0.97-1.53)	0.091
AA	192 (26.70)	364 (25.98)		1.18 (0.92-1.53)	0.199	1.19 (0.92-1.54)	0.192
Additive			0.235	1.08 (0.95-1.23)	0.217	1.09 (0.96-1.23)	0.210
Dominant	563 (78.30)	1,051 (75.02)	0.091	1.20 (0.97-1.49)	0.093	1.21 (0.79-1.50)	0.087
Recessive	527 (73.30)	1,037 (74.02)	0.720	1.04 (0.85-1.27)	0.720	1.04 (0.85-1.27)	0.718

^a^ *χ* ^2^ test for genotype distributions between Kawasaki disease patients and controls. ^b^Adjusted for age and gender.

**Table 3:** Genotype distributions of *IL-1B* gene polymorphisms and susceptibility to coronary artery lesions in Kawasaki disease.

Genotype	CALs (*N* = 312)	NCALs (*N* = 407)	*P* ^a^	Crude OR (95% CI)	*P*	Adjusted OR (95% CI)^b^	*P* ^b^
rs16944							
AA	64 (20.51)	90 (22.11)		1.00		1.00	
AG	154 (49.36)	213 (52.33)		1.02 (0.69-1.49)	0.932	1.01 (0.69-1.49)	0.941
GG	94 (30.13)	104 (25.55)		1.27 (0.83-1.94)	0.269	1.27 (0.83-1.94)	0.277
Additive			0.396	1.40 (0.92-1.40)	0.239	1.13 (0.92-1.40)	0.247
Dominant	248 (79.49)	317 (77.89)	0.604	1.10 (0.77-1.59)	0.604	1.10 (0.76-1.58)	0.616
Recessive	218 (69.87)	303 (74.45)	0.174	1.26 (0.90-1.75)	0.174	1.25 (0.90-1.74)	0.179
rs1143627							
GG	65 (20.83)	91 (22.36)		1.00		1.00	
AG	156 (50.00)	215 (52.83)		1.02 (0.70-1.48)	0.935	1.01 (0.69-1.48)	0.945
AA	91 (29.17)	101 (24.82)		1.26 (0.82-1.93)	0.286	1.26 (0.82-1.93)	0.291
Additive			0.426	1.13 (0.91-1.40)	0.261	1.13 (0.91-1.40)	0.266
Dominant	247 (79.17)	316 (77.64)	0.622	1.09 (0.76-1.57)	0.623	1.09 (0.76-1.57)	0.633
Recessive	221 (70.83)	306 (75.18)	0.192	2.16 (0.77-6.06)	0.146	1.25 (0.89-1.74)	0.194

CALs: coronary artery lesions; NCALs: no coronary artery lesions. ^a^*χ*^2^ test for genotype distributions between Kawasaki disease patients and controls. ^b^Adjusted for age and gender.

**Table 4:** Stratification analysis for the association between rs16944 A>G polymorphism and susceptibility to CALs in Kawasaki disease.

Variables	AA/AG	GG	Crude OR (95% CI)	*P*	Adjusted OR^a^ (95% CI)	*P* ^a^
	CALs/NCALs					
*rs16944*						
Age, month						
≤12	74/98	50/29	**2.28 (1.32-3.95)**	**0.0032**	**2.33 (1.34-4.04)**	**0.0027**
12-60	123/188	37/66	0.85 (0.54-1.36)	0.513	0.86 (0.54-1.37)	0.533
>60	21/17	7/9	0.63 (0.19-2.04)	0.441	0.62 (0.19-2.02)	0.425
Gender						
Females	65/108	28/30	1.55 (0.85-2.83)	0.152	1.47 (0.80-2.69)	0.211
Males	153/195	66/74	1.14 (0.77-1.69)	0.523	1.15 (0.77-1.70)	0.500

^a^Adjusted for age and gender.

**Table 5:** Stratification analysis for the association between rs1143627 A>G polymorphism and susceptibility to CALs in Kawasaki disease.

Variables	GG/AG	AA	Crude OR (95% CI)	*P*	Adjusted OR^a^ (95% CI)	*P* ^a^
	CALs/NCALs					
*rs1143627*						
Age, month						
≤12	74/98	50/29	**2.28 (1.32-3.95)**	**0.0032**	**2.33 (1.34-4.04)**	**0.0027**
12-60	126/191	34/63	0.82 (0.51-1.31)	0.320	0.83 (0.51-1.33)	0.435
>60	21/17	7/9	0.63 (0.19-2.04)	0.441	0.62 (0.19-2.02)	0.425
Gender						
Females	66/108	27/30	1.47 (0.81-2.69)	0.209	1.47 (0.80-2.69)	0.211
Males	155/198	64/71	1.15 (0.77-1.71)	0.140	1.16 (0.78-1.73)	0.460

^a^Adjusted for age and gender.

## Data Availability

The data used to support the findings of this study are available from the corresponding authors upon request.
